# Development of Sustainable Hybrid Cementitious Concrete Using Rice Husk Ash, Quartz Powder, and Bentonite Clay

**DOI:** 10.3390/polym18182227

**Published:** 2026-09-12

**Authors:** Jawad Ahmad, Wael Alattyih

**Affiliations:** 1 Department of Civil and Environmental Engineering, College of Engineering, King Faisal University, Al-Ahsa 31982, Saudi Arabia; 2 Department of Civil Engineering, College of Engineering, Qassim University, Buraydah 51452, Saudi Arabia; w.alattyih@qu.edu.sa

**Keywords:** hybrid cementitious concrete, supplementary cementitious materials, compressive strength, sustainability

## Abstract

This study investigates the performance of hybrid cementitious concrete (HCC) made with rice husk ash (RHA), quartz powder (QP), and bentonite clay (BC). Four concrete mixes, including a control and three HCC mixes containing different proportions of RHA, QP, and BC, were prepared and tested. The properties of the developed concrete were evaluated through slump, density, compressive strength, failure pattern, split tensile strength, water absorption, X-ray diffraction (XRD), scanning electron microscopy (SEM) and energy-dispersive X-ray spectroscopy (EDX). The results indicated that the RHA, QP, and BC reduced workability, with a 13 to 19% reduction compared with the control mixture. Furthermore, the ternary blend HCC-R20-Q20-B20 achieved the highest 28-day compressive strength of 44.6 MPa (22.1% increase compared with the control). Similarly, the highest split tensile strength of 4.56 MPa was obtained by HCC-R30-Q20. Also, the HCC-R30-Q20 exhibited the lowest water absorption value, with a 23.1% reduction. Although the RHA, QP, and BC resulted in a slight decrease in density, all mixes remained within the normal-weight concrete range (2380 to 2408 kg/m^3^). The SEM revealed a denser microstructure, refined pore structure, and improved ITZ. Furthermore, EDX analysis indicated variations in the elemental composition, with variations observed in the Ca/Si and Al/Si ratios.

## 1. Introduction

The construction industry is the largest contributor to global greenhouse gas emissions. Therefore, the construction industry exerts considerable pressure on the environment through intensive extraction of natural resources. Furthermore, it has extensive energy consumption. It contributes to air pollution, climate change, water contamination, and the accumulation of landfill waste. Furthermore, it is also a major source of carbon dioxide (CO_2_) emissions globally [1]. Cement is responsible for 8% of global CO_2_ emissions [2]. A study also indicates that the concentration of carbon dioxide (CO_2_) in the atmosphere has already reached critical levels and increases the risk of serious and irreversible climate change impacts [3]. Therefore, reducing the environmental impact of cement-based materials has become a critical challenge for researchers and engineers. Consequently, the development of sustainable and low-carbon alternative binders has gained attention to mitigate environmental impacts while maintaining the performance required for structural applications.

Geopolymer Concrete (GPC) has attracted research interest in recent decades due to its strong potential to support sustainable development in both industrial and construction sectors [4]. Studies have shown that geopolymer concrete has strong potential to replace conventional concrete due to its superior mechanical strength, enhanced durability, and improved structural performance compared with traditional cement-based materials [5]. It is produced using industrial and agricultural waste. These materials are rich in aluminosilicate compounds. Furthermore, it can be activated through alkaline solutions [6].

Among various agricultural waste materials, rice husk ash (RHA) has been widely recognized as an effective SCM. This is due to its high amorphous silica content, large specific surface area, and strong pozzolanic reactivity. The RHA in cement-based systems has been shown to improve strength development, reduce permeability, and enhance durability performance. Furthermore, it simultaneously contributes to sustainable waste management and circular economy practices. A study [7] indicated that the RHA20 mix demonstrated relatively lower early-age strength. However, it showed enhanced resistance to sulfate attack. These findings indicate its suitability for structures exposed to coastal and sailing environments.

The widespread adoption of geopolymer concrete with fully alkali-activated systems is limited. This may be due to the use of highly alkaline activator solutions such as sodium hydroxide and sodium silicate. The activators can also present challenges related to cost, handling safety, and large-scale field implementation. A study [8] also indicates that the production of geopolymers requires alkaline activators, such as sodium hydroxide and potassium hydroxide, which are highly corrosive and strongly hygroscopic in nature. Therefore, hybrid cementitious concrete (HCC), which combines Portland cement with supplementary aluminosilicate materials, has been proposed as a practical alternative. HCC offers environmental benefits and maintains compatibility with conventional concrete production methods. Hybrid systems allow partial reduction in cement consumption and avoid the complexities associated with highly alkaline chemical activators.

In addition to agricultural ash, the substitution of mineral fillers and nanoscale materials has shown significant potential in improving the microstructure of cementitious mixes. A study [9] indicates that the peak intensity associated with quartz powder is higher than that corresponding to C_3_S and C_2_S, which are the primary phases present in cement. Further, the compressive strength of UHPC initially increases with increasing quartz powder, followed by a decline beyond an optimum dosage level [10]. Also, QP exhibits an effective pore-filling capability in UHPC. The particles of UQP that do not participate in hydration act as a micro-filler, further refining the pore structure and enhancing matrix densification [11]. Therefore, quartz powder (QP) is used as an inert micro-filler due to its ability to enhance particle packing density and reduce pore connectivity. However, a study [12] indicates that the availability of certain SCMs is insufficient to satisfy the increasing demand of the construction industry. Therefore, there is a growing need to explore alternative and widely available materials that can contribute to sustainable binders.

Bentonite clay (BC) is a soft and easily mouldable clay formed through the weathering and decomposition of volcanic tuff and ash deposits [13]. The bentonite clay and quarry dust reduce the concrete workability compared to the control mix. Furthermore, quarry dust shows the lowest workability due to the relatively coarser and angular particles [14]. Furthermore, a study [15] indicates that the absence of cement resulted in a reduction in strength (compressive and tensile strength). It also suggests that pozzolanic materials cannot be used alone as binding materials [15].

Therefore, the combined utilization of RHA, QP, and BC is expected to produce synergistic effects through simultaneous pozzolanic reaction, micro-filler densification, and nano-scale modification of the mix. Numerous studies have investigated the use of SCM in concrete. However, most previous research has focused on binary binder systems with single pozzolanic materials such as fly ash, slag, or silica fume. However, limited research has explored the combined influence of agricultural waste ash, mineral filler, and nano-clay materials in hybrid cementitious systems.

The novelty of the present study lies in the multi-scale modification of an OPC-based HCC system. Geopolymer systems generally require highly alkaline activating solutions. However, a previous study [8] indicates that the production of geopolymers requires alkaline activators which are highly corrosive and strongly hygroscopic in nature. The proposed system maintains a conventional cement-based production approach without external alkaline activators. The interaction between RHA, QP, and BC in ternary binder systems and their collective influence on microstructural development and mechanical performance remain insufficiently understood. Furthermore, previous investigations often examined filler effects or pozzolanic reactions independently. The combined pozzolanic reaction effect or filler effect of RHA, QP, and BC has received limited attention in the literature.

Therefore, this study aims to develop sustainable HCC made with rice husk ash, quartz powder, and bentonite clay as a partial replacement of cement. The research investigates the performance of binary and ternary binder systems with constant binder content and water-to-binder ratio. The research develops a multi-scale modified hybrid binder mix without highly alkaline activator solutions. The findings of this study are expected to contribute to the advancement of environmentally friendly concrete materials with reduced cement consumption and improved long-term performance.

## 2. Experimental Setups

### 2.1. Materials

**Figure 1:**
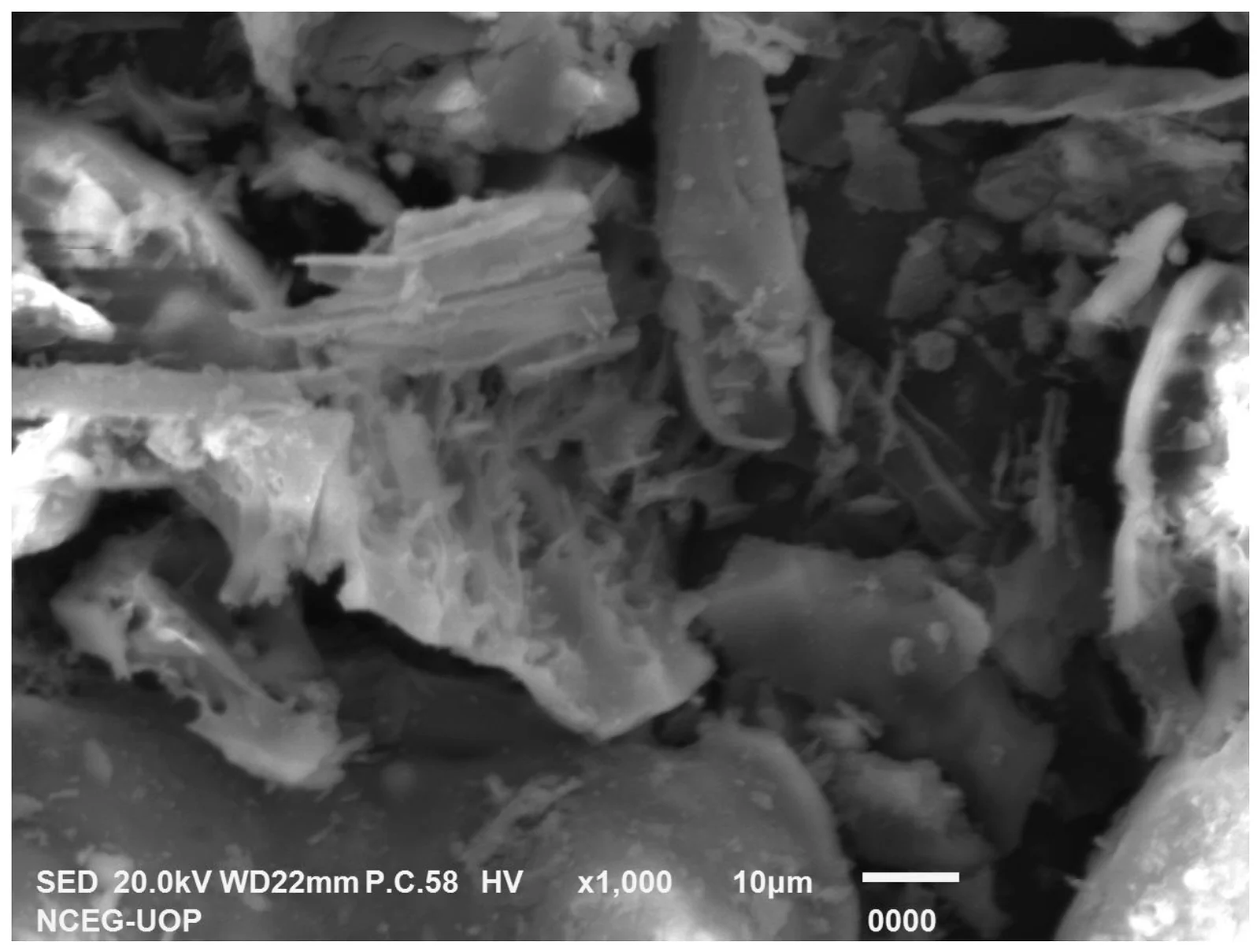
SEM of rice husk ash particles.

Ordinary Portland Cement (OPC) in accordance with ASTM C150 was used as the binder in the control mixture. Rice husk ash (RHA) was used as a sustainable SCM. The RHA was gray in color, possessed a low specific gravity (2.14), and had a fine particle size less than 75 microns. The SEM of the RHA particles shows porous morphology with a cellular structure and numerous visible voids (Figure 1). The porous surface provides a relatively high surface area and can promote interaction with the cementitious mix. However, the porous structure of RHA can increase water demand due to the absorption of water.

**Figure 2:**
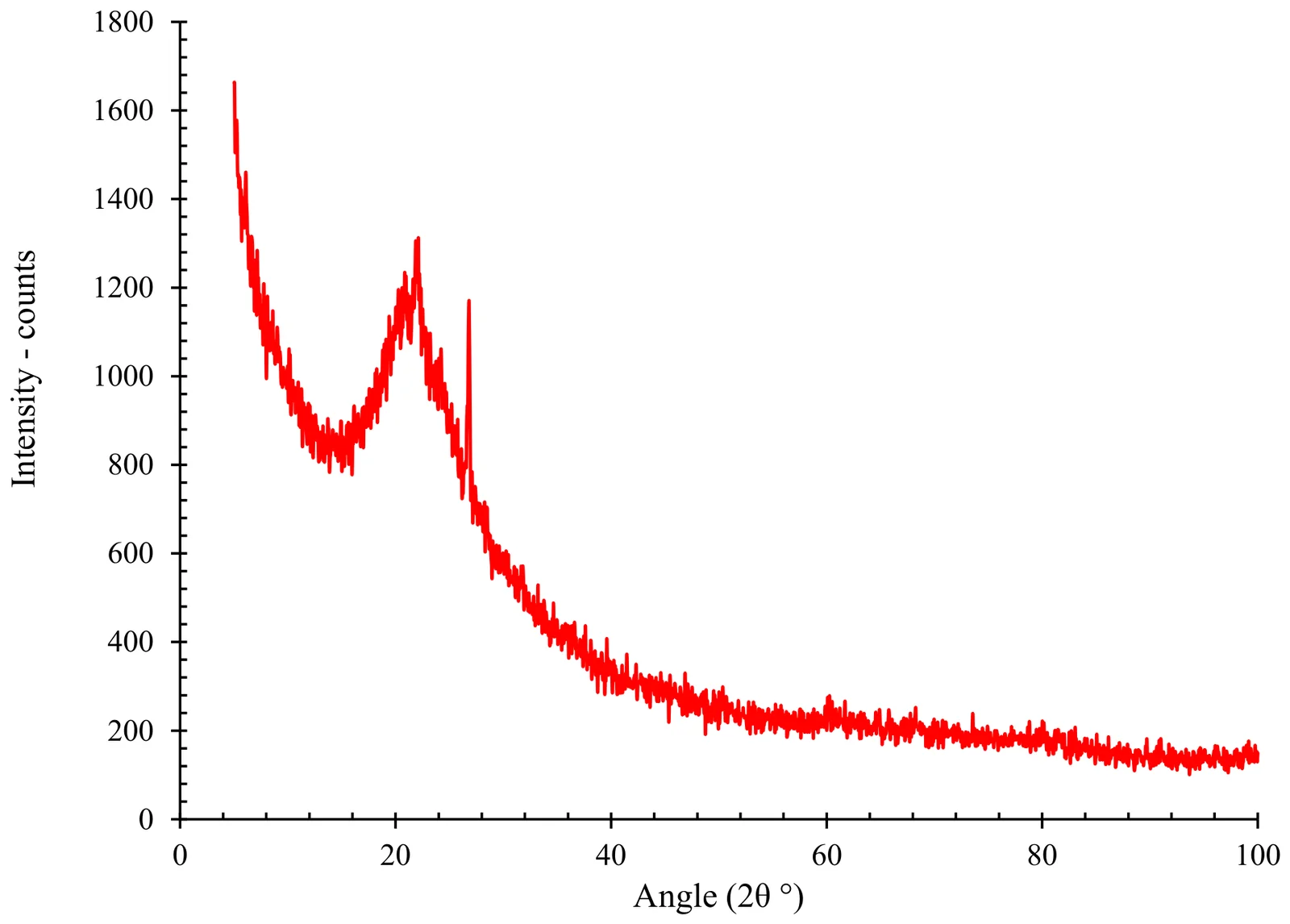
XRD of rice husk ash.

The diffractogram is a typical characteristic of an amorphous nature (Figure 2). The amorphous phase is highly desirable for cementitious applications because it enhances pozzolanic reactivity. A minor diffraction peak observed near 26.8° (2θ) can be crystalline quartz. Therefore, it suggests the presence of a small amount of residual crystalline silica. However, the relatively low intensity of this peak compared with the broad amorphous hump suggests that the RHA is largely amorphous. The high amorphous silica content enables RHA to react readily with the released calcium hydroxide, leading to the formation of additional CSH gel, which contributes to densification and improved mechanical properties. Therefore, the XRD results suggest the suitability of the produced RHA as a suitable SCM.

**Figure 3:**
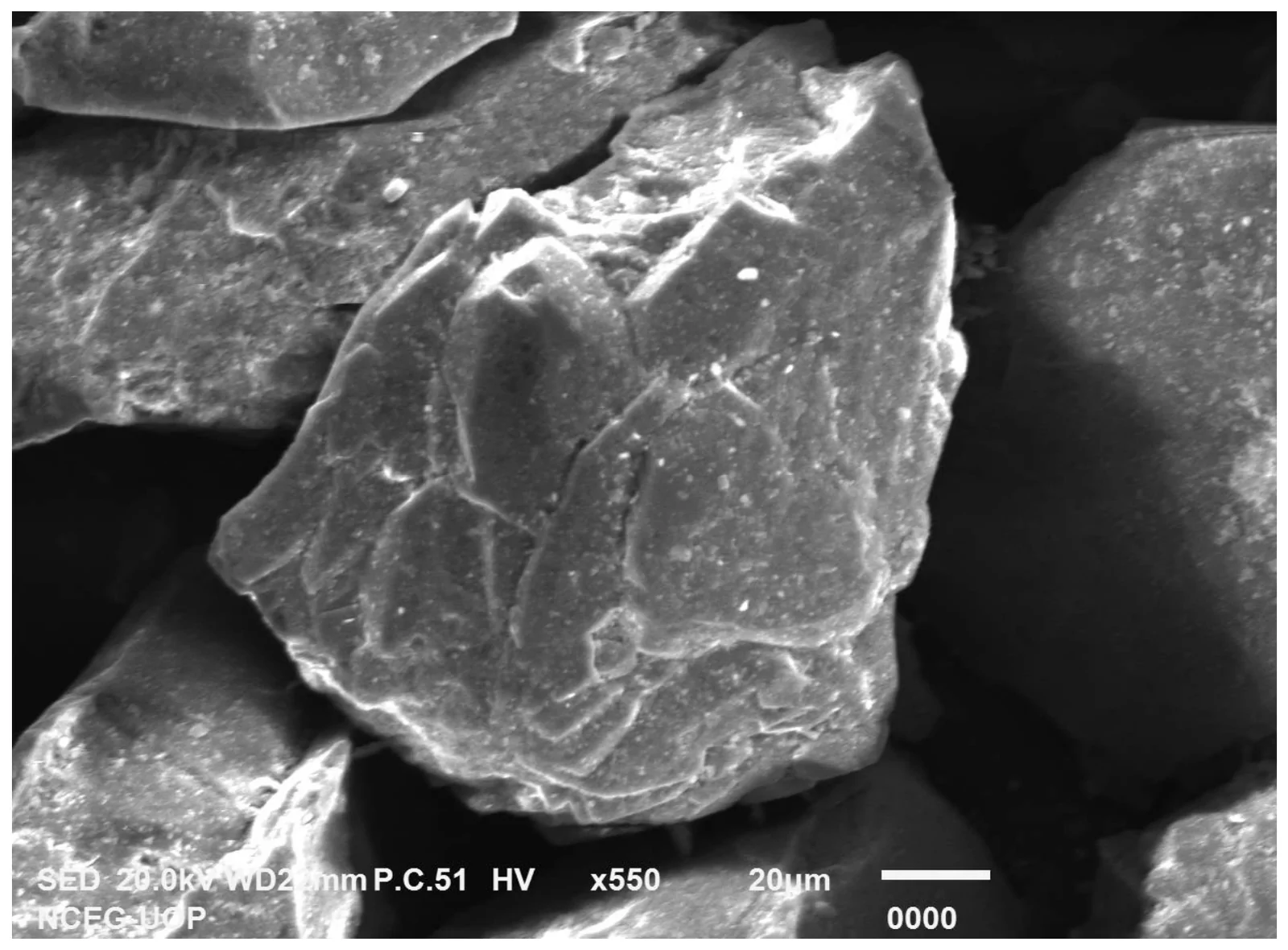
SEM of quartz powder particles.

Quartz powder (QP) was used as a micro-filler to improve particle packing. The SEM of QP shows an irregular and angular morphology with sharp edges (Figure 3). The irregular and angular morphology can enhance particle packing and filling of voids within the cementitious mix, which leads to a denser microstructure.

**Figure 4:**
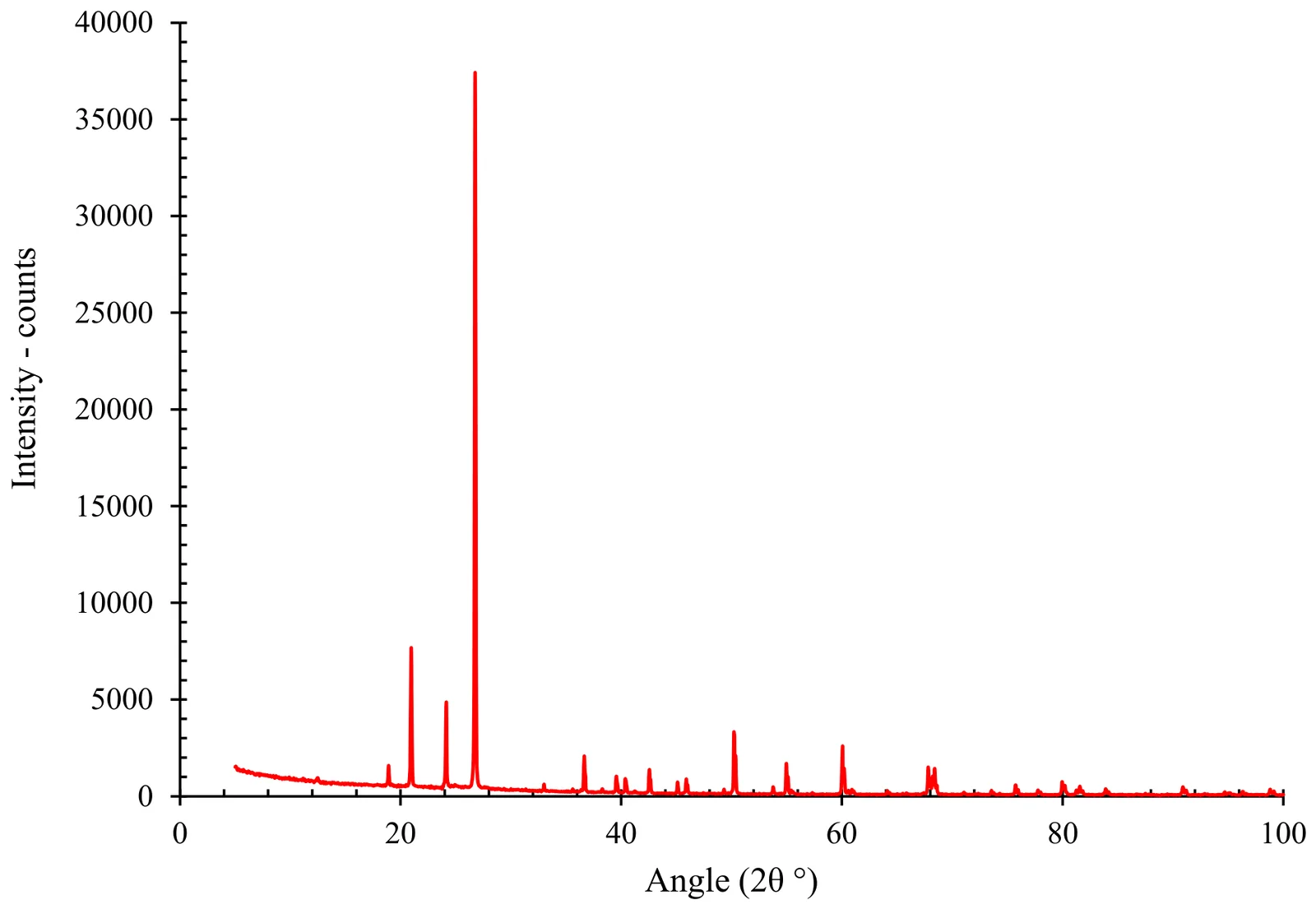
XRD of quartz powder.

The XRD pattern is characterized by a series of intense diffraction peaks (Figure 4) and suggests a crystalline nature. The most prominent peak appears at 26.7° (2θ), which can be the characteristic reflection of crystalline quartz (SiO_2_). The high crystallinity of quartz powder can suggest its limited pozzolanic reactivity. However, its ultrafine particles can effectively act as a micro-filler and improve particle packing. Therefore, quartz powder contributes to mix densification and enhancement of the mechanical properties.

**Figure 5:**
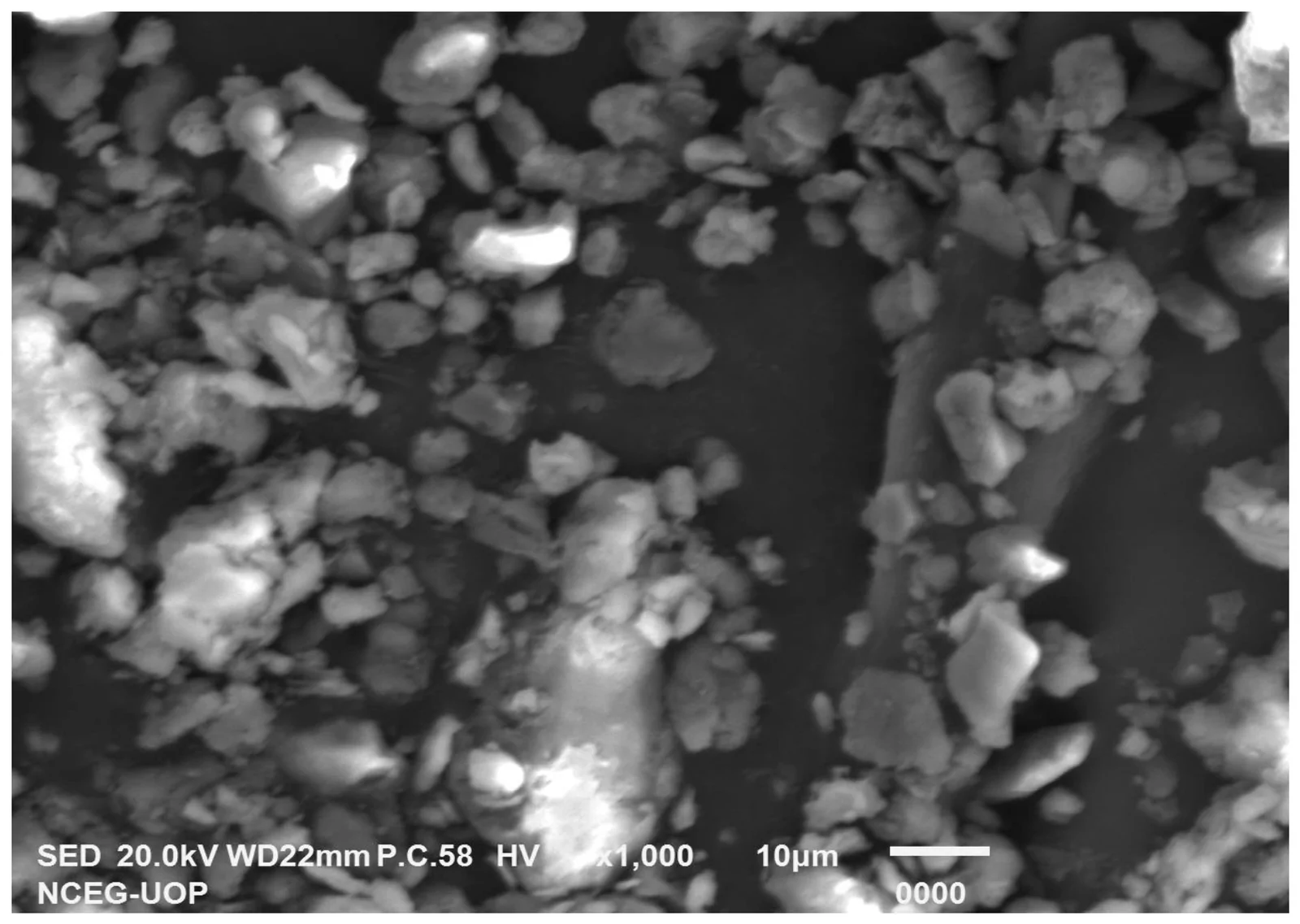
SEM of bentonite clay particles.

Furthermore, bentonite clay (BC) was used to enhance pore refinement and microstructural development. The SEM image of BC shows mostly rounded to sub-rounded particles (Figure 5). Noticeable particle agglomeration is also observed, with fine particles clustered around larger particles. The fine particle size can enhance particle packing and microstructural refinement due to filling voids.

**Figure 6:**
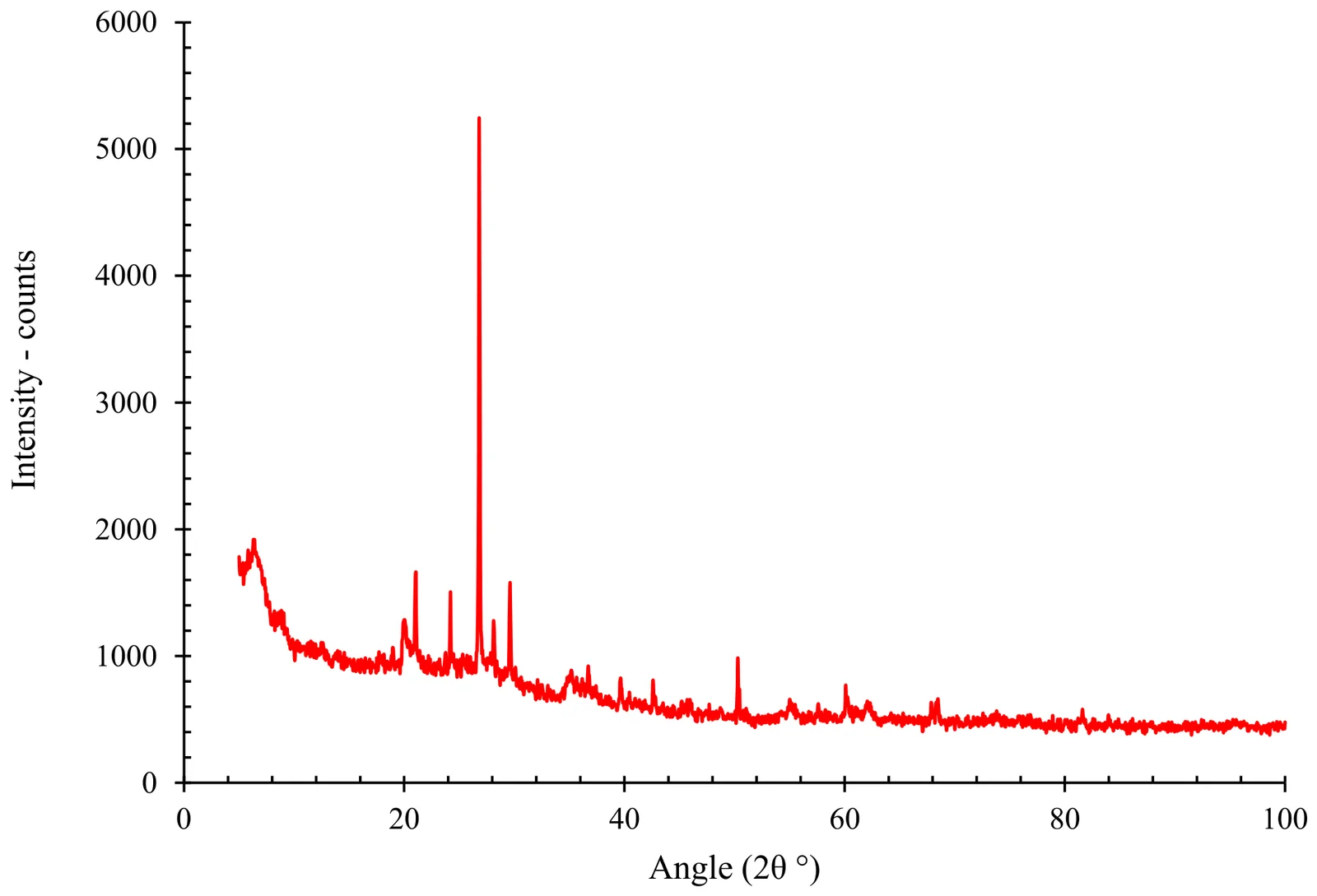
XRD of bentonite clay.

The XRD pattern (Figure 6) shows a prominent diffraction peak at 26.8° (2θ), which can be characteristic of crystalline quartz (SiO_2_). The intensity of the peak suggests that quartz is the dominant crystalline phase present. Furthermore, several minor peaks were detected at higher diffraction angles. The presence of silica suggests pozzolanic potential. The pozzolanic reaction can promote the formation of additional calcium silicate hydrate (CSH) gel. Therefore, CSH gels lead to improved matrix densification and enhanced mechanical performance.

River sand obtained from a natural source served as the fine aggregate in all concrete mixes with particles passing through a 4.75 mm sieve. It possessed a specific gravity of 2.63, water absorption of 1.4%, and a fineness modulus of 2.3. Crushed coarse aggregate with a nominal maximum particle size of 19.5 mm was utilized in the concrete mixes. The coarse aggregate shows a specific gravity of 2.85, water absorption of 0.8%, and an angular particle shape. Potable water was used for mixing and curing the concrete specimens. A superplasticizer was added to achieve the desired workability.

### 2.2. Mix Design of HCC

Four concrete mixes were designed in this study to investigate the combined effect of RHA, QP, and BC in HCC. The individual effects of RHA, QP, and BC have been extensively investigated in previous studies. Therefore, the present research focused on selected binary and ternary combinations to evaluate their contribution to the concrete performance. The total binder content was constant at 420 kg/m^3^ for all mixes. Cement was partially replaced with varying proportions of RHA, QP, and BC to investigate the combined effect on the mechanical and durability performance of concrete. The control mixture consisted of 420 kg/m^3^ cement, 550 kg/m^3^ natural river sand, 1020 kg/m^3^ coarse aggregate, 170 kg/m^3^ water, and 2.1 kg/m^3^ superplasticizer. The w/b ratio was fixed at 0.40 for all mixes to maintain consistent conditions among the mixes. In mix HCC-R30-B20, cement was partially replaced with 30% RHA (126 kg/m^3^) and 20% bentonite (84 kg/m^3^), resulting in 210 kg/m^3^ cement. Bentonite clay has fine particle characteristics that can enhance particle packing density and improve the ITZ. The aggregate was kept constant at 550 kg/m^3^ natural river sand and 1020 kg/m^3^ coarse aggregate. Furthermore, the water and superplasticizer were constant at 170 kg/m^3^ and 2.1 kg/m^3^, respectively. For mixture HCC-R30-Q20, cement was replaced with 30% RHA (126 kg/m^3^) and 20% quartz powder (84 kg/m^3^). Quartz powder was used as an inert micro-filler to enhance packing density and reduce pore connectivity. Therefore, it improves strength development and durability performance. The quantities of aggregates, water, and superplasticizer remained constant. Therefore, the variations in performance were primarily attributed to binder modification. In mix HCC-R20-Q20-B20, a ternary binder system was adopted in which cement was replaced with 20% RHA (84 kg/m^3^), 20% QP (84 kg/m^3^), and 20% BC (84 kg/m^3^), with cement at 40% (168 kg/m^3^). The combination was designed to investigate the potential beneficial interaction among pozzolanic reactions and refined microstructure. The detailed quantification of mix proportions for all mixes is presented in Table 1.

### 2.3. Mixing and Casting

Control and HCC mixes were prepared in a laboratory mixer. First, the coarse aggregate, natural river sand, cement, RHA, QP, and BC were dry mixed for 3 to 4 min to ensure a uniform distribution of the dry constituent materials. Subsequently, water mixed with the required dosage of superplasticizer was gradually added to the dry mixture, and continuous mixing was maintained. The mixing process continued for an additional 4 to 5 min until a homogeneous and workable concrete mixture was obtained. After mixing, the fresh concrete was tested for workability and then placed into cylindrical molds in three equal layers. Each layer was compacted using a vibrating table to remove entrapped air and ensure proper consolidation. The top surface of the specimens was finished and levelled using a steel trowel. The specimens were kept at room temperature for 24 h. After demolding, the specimens were cured in water at a temperature of 25 ± 2 °C until the designated testing ages. The cured specimens were subsequently used for density, compressive strength, split tensile strength, and water absorption tests. Three samples for each mix were tested, and the average value was considered the result. The limited sample size may restrict the statistical validity and generalizability of the observed trends. Further studies with larger datasets are recommended to validate the findings.

### 2.4. Experimental Testing

The fresh and hardened properties of each mix were evaluated through slump, density, compressive strength, split tensile strength, and water absorption tests. All tests were conducted as per relevant ASTM standards. Three specimens were tested for each mix, and the average value was reported as the result. The testing standards and experimental details are summarized in Table 2.

A broken sample was collected from the cut surface of the cylindrical specimen during testing for SEM analysis. The sample was carefully cut and polished to obtain a suitable surface for SEM examination. The prepared specimen was dried and subjected to SEM analysis to investigate the microstructure and ITZ. Also, EDX was performed to determine the elemental composition of the selected concrete samples. The analysis was conducted using a normalized analysis procedure, with three iterations.

## 3. Results and Discussion

### 3.1. Slump

**Figure 7:**
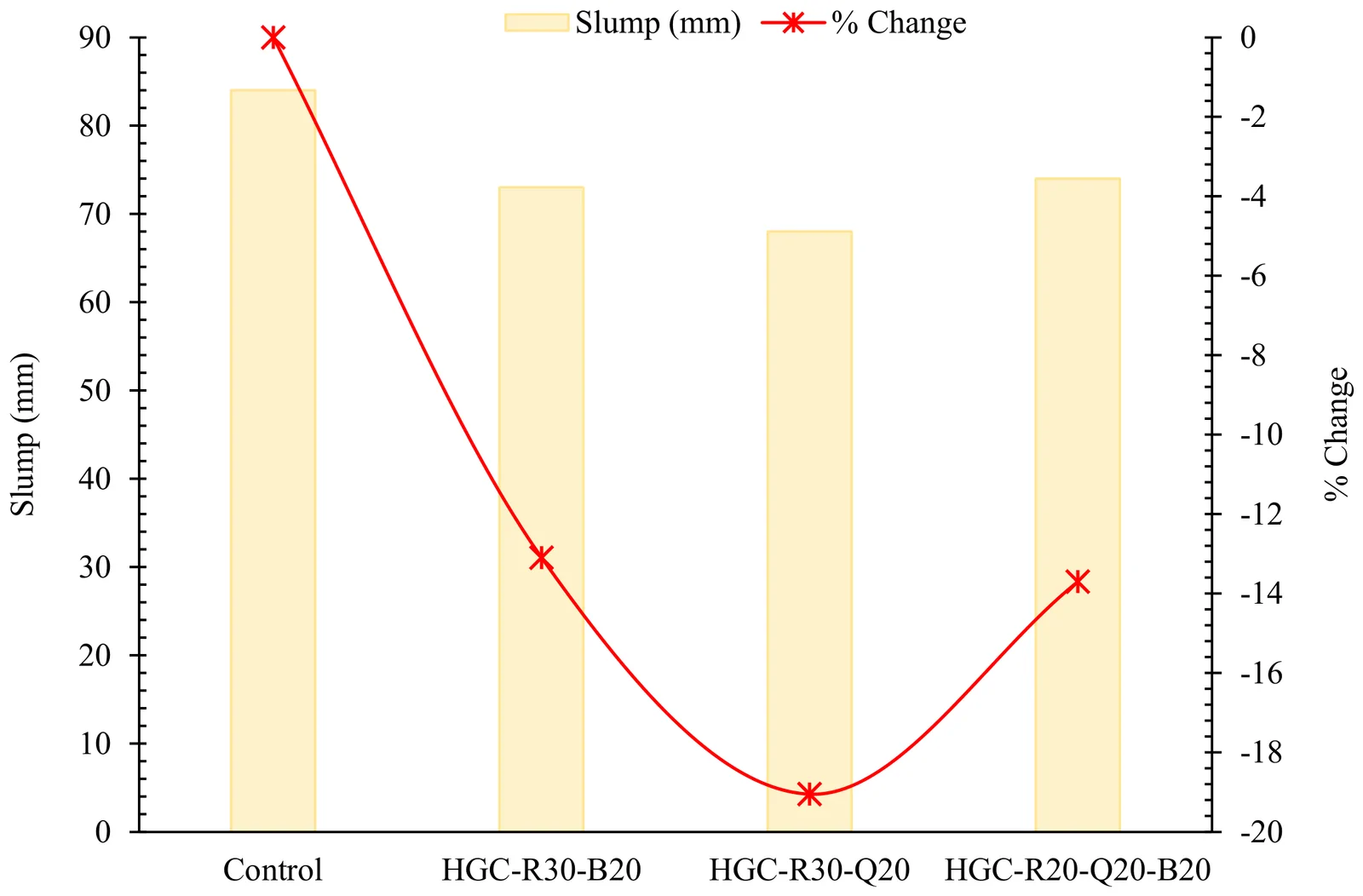
Slump test results of the control and HCC mixes.

The slump results for concrete made with RHA, QP, and BC indicated reduced workability compared with the control mix (Figure 7). The control mixture shows the highest slump value of 84 mm, which indicates adequate workability. In contrast, all modified mixtures showed lower slump values, ranging from 68 to 73 mm, with reductions of 13 to 19% compared to the control. The mixture HCC-R30-B20 recorded a slump of 73 mm with a 13.1% decrease compared with the control. The reduction can be attributed to the high specific surface area and water absorption capacity of bentonite clay. Therefore, it increased the water demand of the mixture and reduced the availability of free water for lubrication. The presence of nano-sized clay particles also promoted particle flocculation, leading to increased internal friction and lower flowability. A further reduction was observed for HCC-R30-Q20, which shows the lowest slump value of 68 mm with a 19.0% decrease. Although quartz powder acts mainly as an inert filler, its fine particle size increases the total surface area of the binder system. However, it requires additional water to maintain flowability. The ternary blend HCC-R20-Q20-B20 achieved a slump of 74 mm, a 13.7% reduction relative to the control. Although the binary mixture contained both QP and BC, the lower RHA replacement level (20%) partially mitigated the loss of workability. The interaction between the pozzolanic RHA, micro-filler QP, and fine particles of BC can produce a cohesive mix with acceptable consistency and dense particle packing. The observed reduction in slump is consistent with previous studies, which report decreased flowability in concrete with RHA [20], QP [21], and BC [22]. The decrease is due to their increased water demand and particle interaction effects.

### 3.2. Compressive Strength

**Figure 8:**
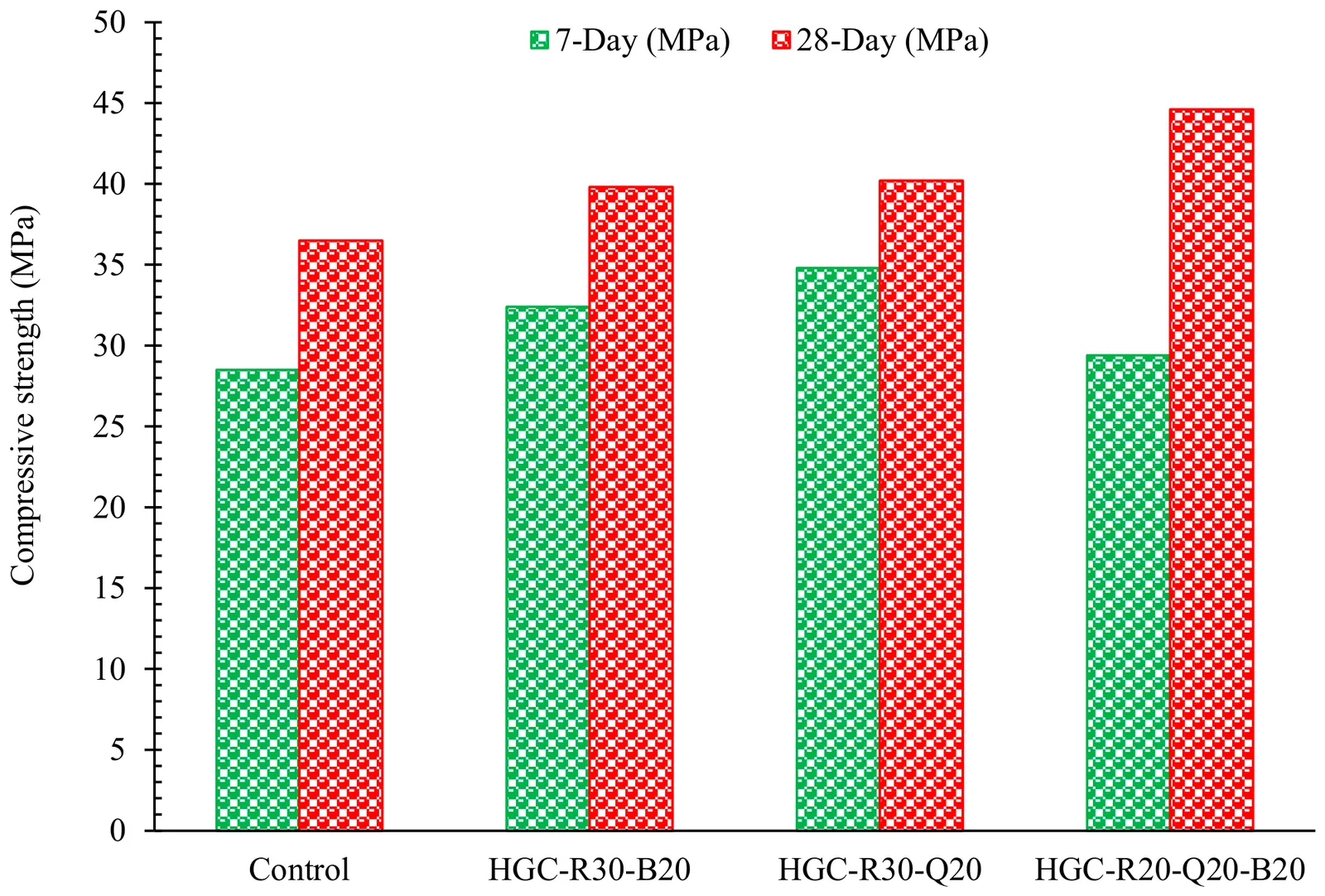
Compressive strength of the control and HCC mixes.

The compressive strength results of the control and HCC at 7 and 28 days are presented in Figure 8. The results demonstrate that RHA, QP, and BC influenced the strength of the concrete mixes. The findings indicate that the control mix achieved a compressive strength of 28.5 MPa at 7 days. The mixes made with supplementary materials enhanced strength, with HCC-R30-B20 and HCC-R30-Q20 attaining strengths of approximately 32.2 MPa and 34.8 MPa, with increases of 12.9% and 21.1%, respectively, compared with the control. The improvement can be attributed to the pozzolanic activity of RHA and the filler effect of QP and BC. Therefore, it promotes mixed densification and accelerated strength development. The ternary mixture HCC-R20-Q20-B20 shows a 7-day strength of 29.4 MPa, which was slightly higher than that of the control but lower than that of the binary mixtures. This is due to the higher cement replacement (60%), which delayed early hydration reactions. Furthermore, the control mix shows a compressive strength of 36.5 MPa, and HCC-R30-B20 and HCC-R30-Q20 achieved strengths of 39.8 MPa and 40.2 MPa with increases of 9.0% and 10.1%, respectively. Previous findings also demonstrate that cement replacement up to 30% with RHA can be successfully utilized and maintain satisfactory strength development and permeability characteristics of concrete [23]. Similarly, previous findings indicated that bentonite clay resulted in a noticeable enhancement in strength performance [24]. The increase is attributed to the continued pozzolanic reaction of RHA. The pozzolanic reaction consumed calcium hydroxide and generated additional CSH gel. The CSH gel led to a denser microstructure.

Furthermore, the micro-filler action of quartz powder and the pore-refining capability of bentonite contributed to improved particle packing. Therefore, the interfacial transition zone (ITZ) improved, and the mix showed better strength. Furthermore, the highest 28-day compressive strength was obtained by the HCC-R20-Q20-B20 mixture, reaching 44.6 MPa, with a 22.1% increase compared with the control mixture. The improvement in concrete compressive strength is due to the synergistic interaction between RHA, QP, and BC. The pozzolanic activity of RHA, combined with the filler effect of QP and the finer particle characteristics of bentonite, produced a highly compact and refined microstructure. Therefore, the HCC-R20-Q20-B20 mix had reduced porosity and enhanced load-transfer capacity.

**Figure 9:**
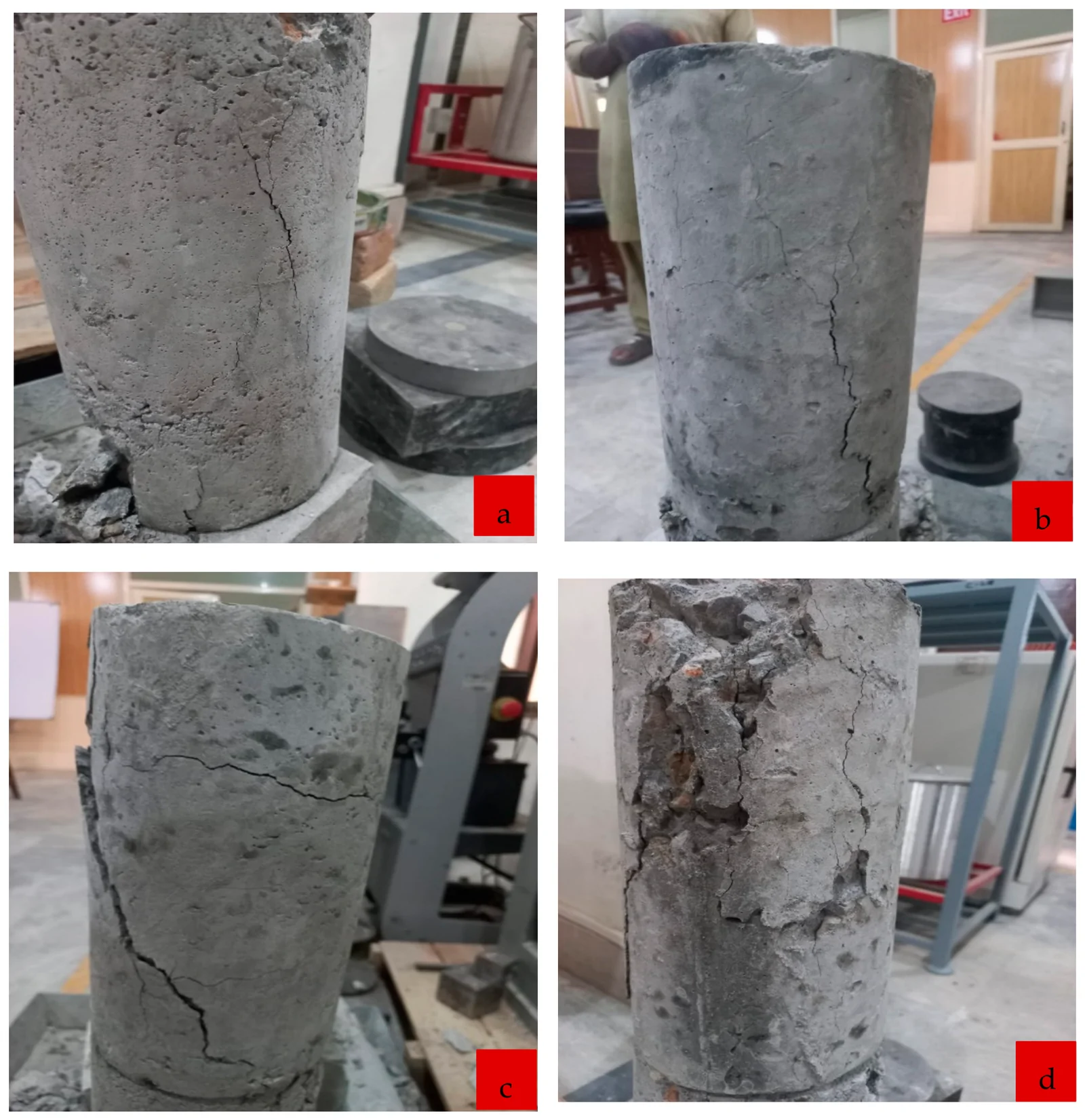
Failure pattern of the control and HCC mixes: (**a**) Control, (**b**) HCC-R30-B20, (**c**) HCC-R30-Q20 and (**d**) HCC-R20-Q20-B20.

Figure 9 shows the failure patterns of the control and HCC specimens subjected to compressive loading. The findings indicate that the control specimen shows a splitting failure. The splitting failure is characterized by the formation of a dominant longitudinal crack extending along the height of the cylinder. Noticeable crack widening and localized surface deterioration were observed. Therefore, this can indicate a relatively brittle failure mechanism. Furthermore, the HCC-R30-B20 specimen also displayed a similar longitudinal cracking pattern. However, the HCC-R30-Q20 specimen exhibited a combination of longitudinal and inclined cracks with limited surface damage. The development of multiple crack paths can suggest a more uniform stress distribution. The improved particle packing and refined microstructure enhanced the load-transfer capacity and resistance to crack growth. In contrast, the HCC-R20-Q20-B20 ternary blend shows more severe cracking accompanied by localized crushing and surface spalling. The failure was characterized by the formation of multiple interconnected cracks and partial loss of material near the failure zone. Despite better compressive strength, the specimen showed a more abrupt failure pattern. This can be attributed to its compact and dense microstructure. The enhanced densification increased the load-carrying capacity, but once the peak load was reached, the stresses resulted in localized cracking.

### 3.3. Split Tensile Strength

**Figure 10:**
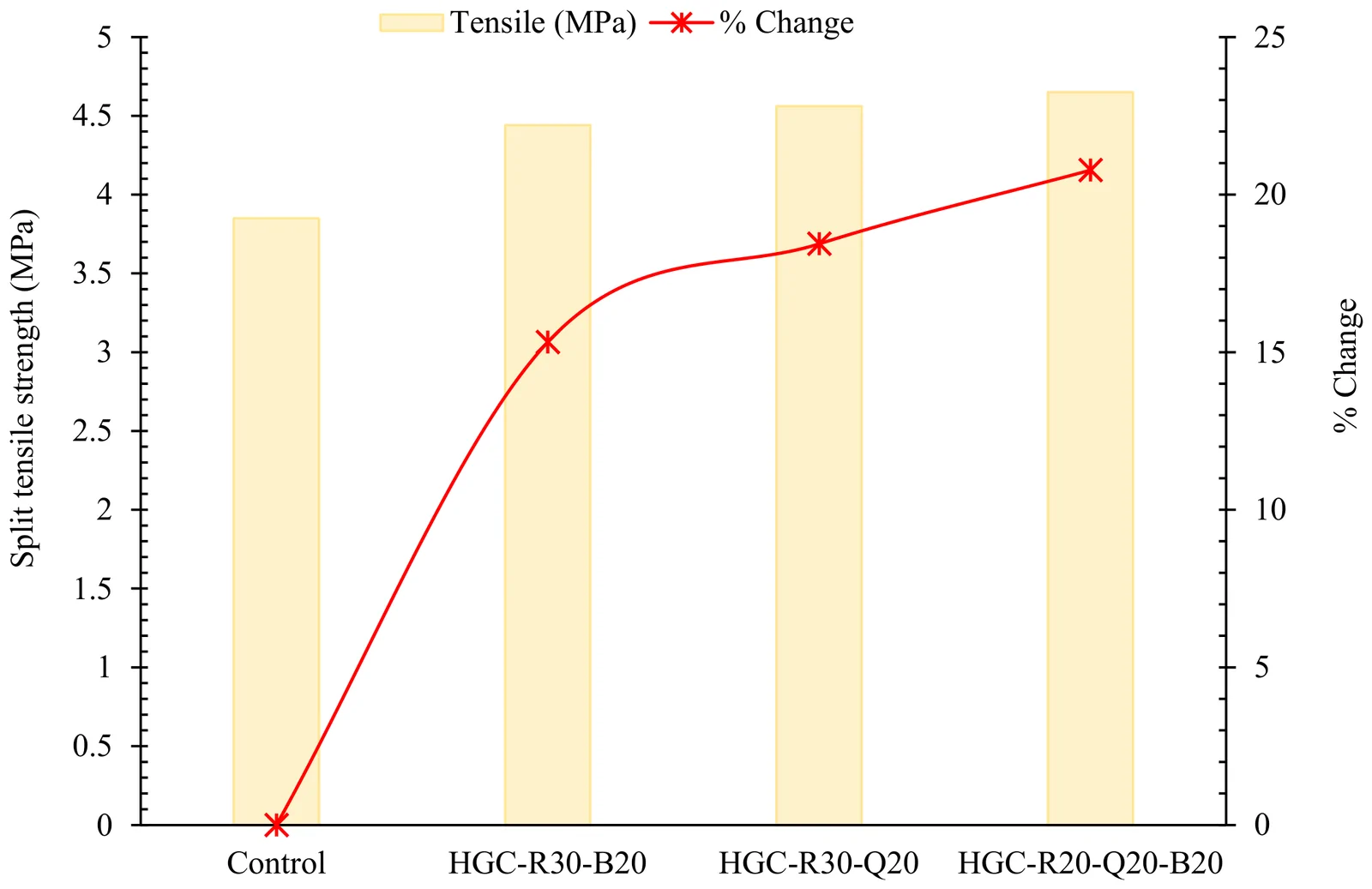
Split tensile strength of the control and HCC mixes.

The split tensile strength results of the control and HCC mixes at 28 days are presented in Figure 10. The control mix shows a split tensile strength of 3.85 MPa. In contrast, the binary blends demonstrated notable improvements, with HCC-R30-B20 and HCC-R30-Q20 achieving strengths of 4.44 MPa and 4.56 MPa, with increases of 15.3% and 18.4%, respectively, compared to the control. The improvement can be attributed to the combined effects of the reactive silica present in RHA and the micro-filler action of QP and BC. The pozzolanic reaction of RHA promotes the formation of additional binding gels. The CSH gel leads to improved matrix densification and a stronger interfacial transition zone (ITZ). Moreover, the ultrafine particles of quartz powder effectively fill micro voids within the matrix and improve particle packing. Therefore, it reduces stress concentrations under tensile loading. Similar improvements in tensile strength were reported for blended cementitious mixes made with supplementary materials. Furthermore, the HCC-R30-Q20 shows the highest split tensile strength, which indicates that the synergistic interaction between RHA and QP was particularly effective in enhancing crack resistance and tensile load-transfer capacity. This can be attributed to the nucleation effect of finely ground quartz particles, which accelerates the formation of hydration products and contributes to a more homogeneous and compact microstructure. The ternary blend HCC-R20-Q20-B20 achieved a split tensile strength of 4.12 MPa, with an increase of 7.0% compared with the control mixture. Although its tensile strength remained higher than that of the control, it was lower than that of the binary blends. The reduction can be associated with the higher replacement level of cement and could have limited the availability of reactive constituents required for optimal gel formation. Therefore, the ternary system still provided a denser matrix than the control concrete and maintained an improvement in tensile performance as compared to the control mix.

### 3.4. Water Absorption

**Figure 11:**
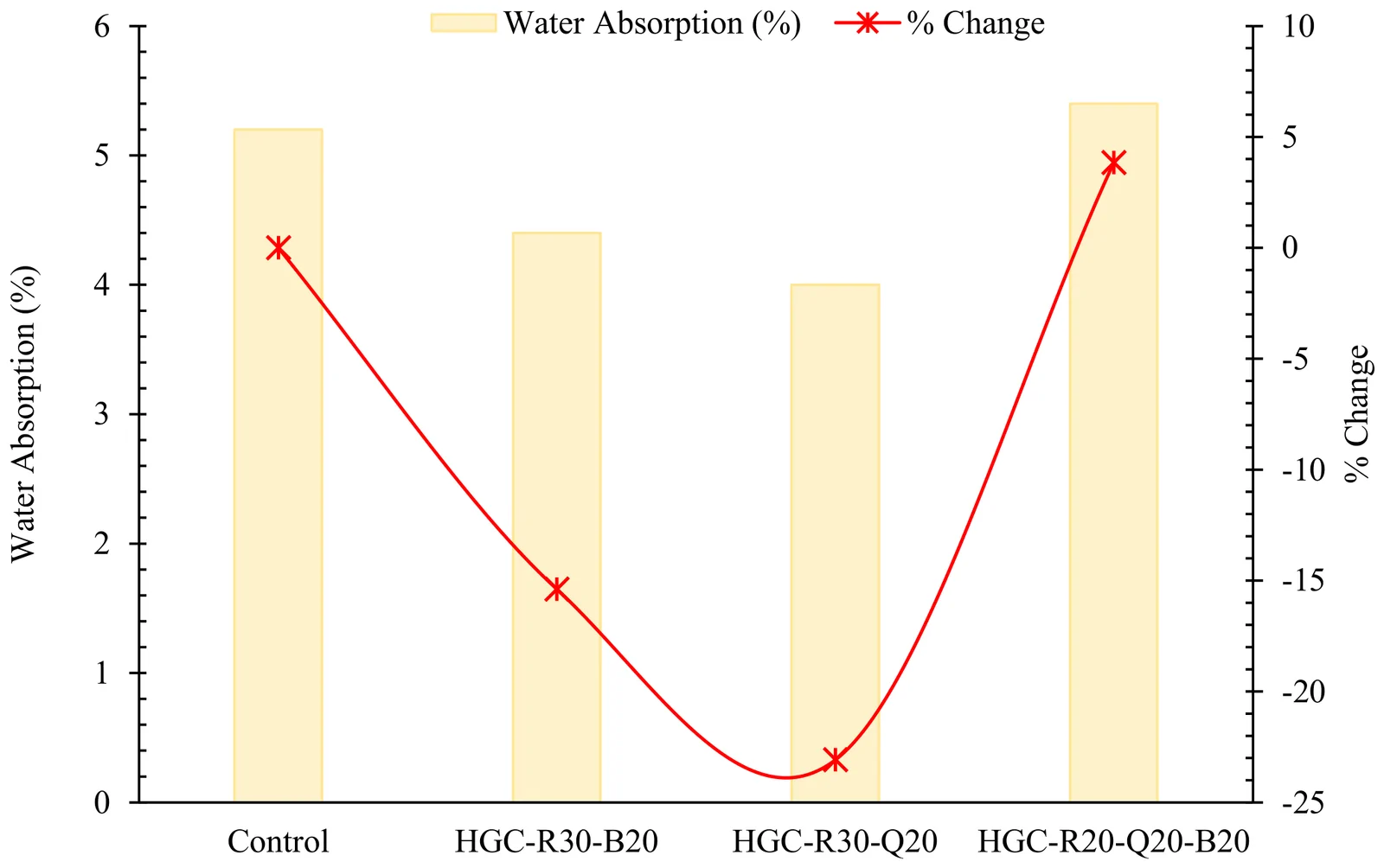
Water absorption of the control and HCC mixes.

The water absorption results of the control and HCC mixes are presented in Figure 11. The mix made with RHA, QP, and BC affected the permeability-related properties of the concrete. The control mix shows a water absorption value of 5.2%. The mix made with SCM reduced water absorption, which indicates an improvement in the pore structure and compactness. The mix made with HCC-R30-B20 achieved a water absorption of 4.4%, which represents a reduction of 15.4% compared with the control mixture. The improvement is due to the pozzolanic activity of RHA and the filler effect of BC, which refined the pore structure and reduced the connectivity of capillary pores. The formation of additional reaction products contributed to matrix densification and limited water penetration. A further reduction in water absorption was observed for HCC-R30-Q20 (lowest value of 4.0%), with a decrease of 23.1% relative to the control concrete. The superior performance of the mixture is due to the combined effect of the highly reactive silica in RHA and the micro-filler action of quartz powder. The ultrafine quartz particles effectively filled micro voids and enhanced particle packing density, resulting in a more compact microstructure with reduced porosity and lower permeability. Therefore, the movement of water through the concrete matrix was significantly restricted. In contrast, the ternary blend HCC-R20-Q20-B20 shows a water absorption value of 5.4%, which was 3.8% higher than that of the control mixture. Although the combined use of RHA, QP, and BC can contribute to microstructural refinement, the relatively high replacement level of cement adversely affected the continuity of the cementitious mix, leading to an increase in pore volume and water-accessible voids. The ternary mixture did not show an improvement in water absorption compared with the control mix and other mixes. However, water absorption alone is not sufficient to establish an improvement in durability. Further durability-related tests are required to comprehensively assess the durability performance of the mixes.

### 3.5. Density

**Figure 12:**
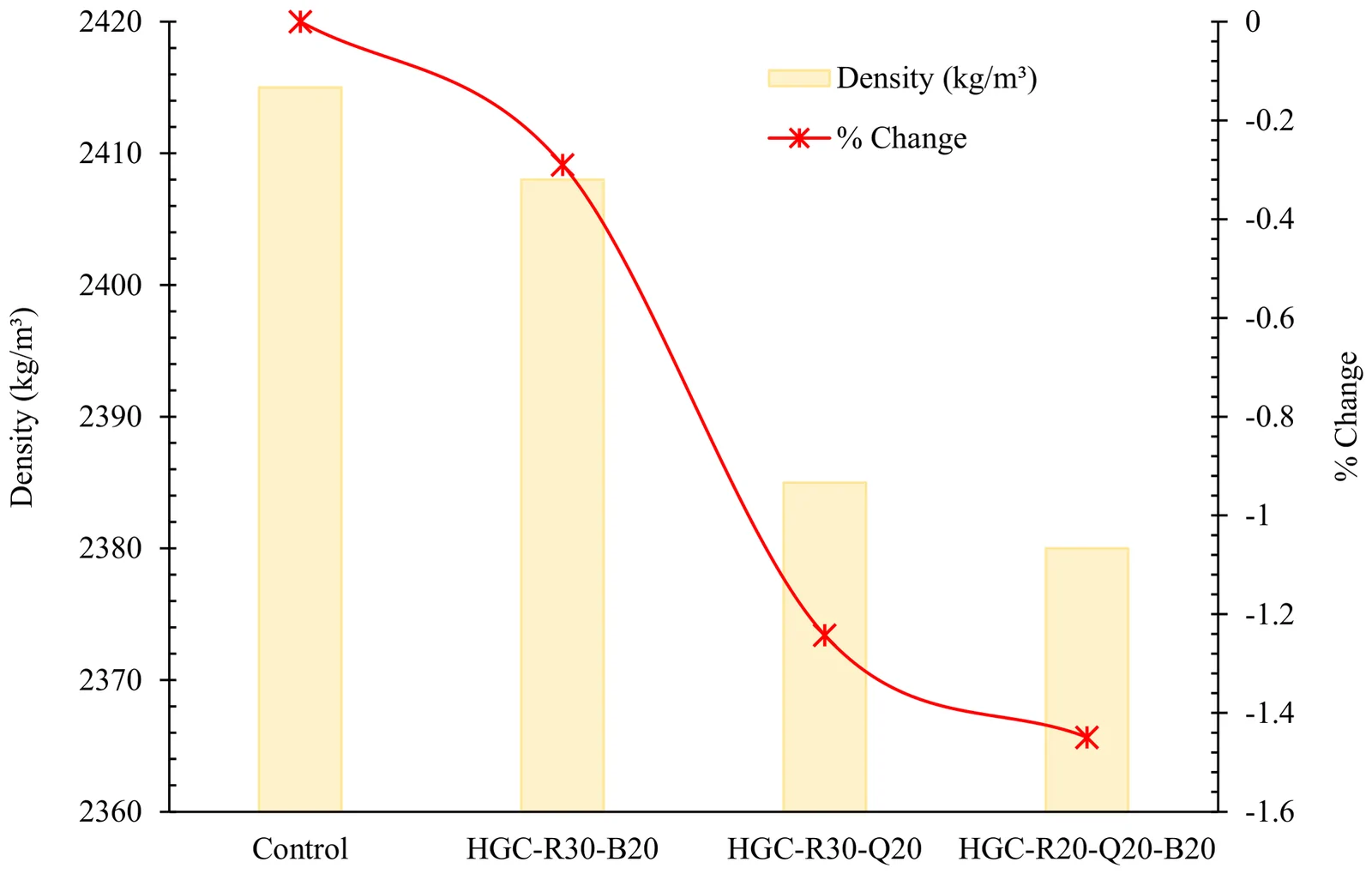
Density of the control and HCC mixes.

The density results of the control and HCC mixes are shown in Figure 12. The control mix shows the highest density of 2415 kg/m^3^. The mix made with RHA, QP, and BC resulted in a slight reduction in density as compared to the control. A previous study also indicates that the density, specific gravity, and average particle size of RHA were lower than cement [23]. The density of HCC-R30-B20 was 2408 kg/m^3^. Similarly, the HCC-R30-Q20 shows a density of 2385 kg/m^3^. QP contributes to mix densification through its filler effect. However, the replacement of cement with lower-density materials resulted in a decline in the bulk density. However, the findings suggest that the improved packing characteristics of the finely divided particles effectively minimized the loss in density. Furthermore, the HCC-R20-Q20-B20 ternary mix shows the lowest density (2380 kg/m^3^). The reduction is due to the higher replacement of cement with the relatively lower specific gravity of RHA and BC. However, the decrease was small, which indicates that the synergistic interaction among the supplementary materials maintained a compact internal structure. Therefore, the findings suggest that the developed HCC can maintain density comparable to conventional normal-weight concrete.

### 3.6. Scanning Electron Microscopy (SEM) Analysis

**Figure 13:**
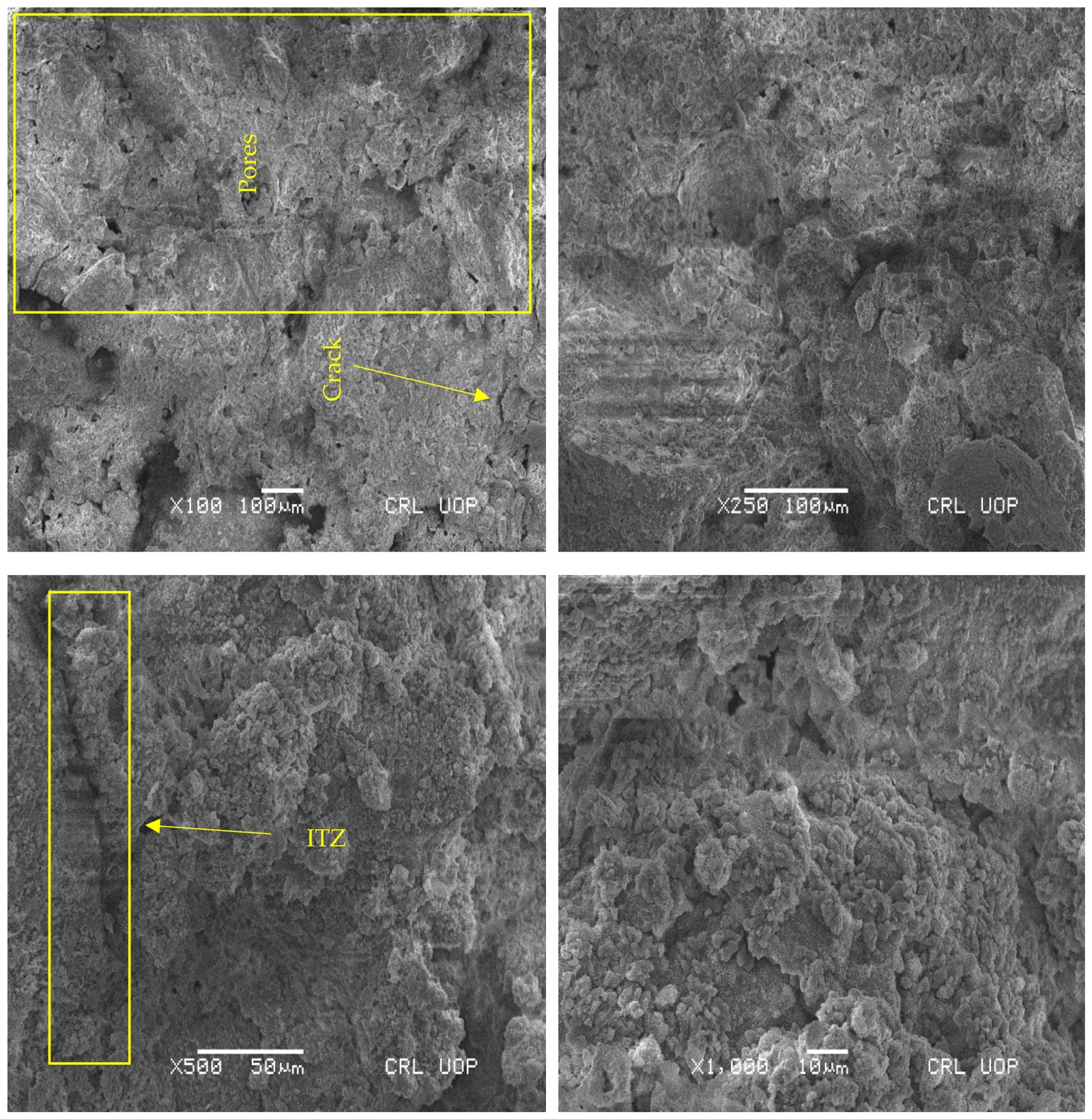
SEM of control mix.

The SEM of the control mix reveals a relatively homogeneous microstructure (Figure 13). However, the presence of small pores, microcracks, and a distinguishable ITZ was observed. The mix shows irregularly distributed voids and microcracks. The presence of microcracks also suggests the development of internal stresses caused by cement hydration, shrinkage effects, and moisture loss. The microcracks may act as preferential pathways for acid or water and potentially influence long-term durability performance. The observations provide a baseline for comparison with modified HCC, which enhanced particle packing density and improved ITZ quality. However, the SEM observations are based on selected regions of the specimens (central part of crushed cylinder during compressive strength) and are therefore considered qualitative. No quantitative image analysis of porosity, pore-size distribution, crack density, or ITZ thickness was performed in this study. Accordingly, the observed differences should not be interpreted as quantitative evidence of porosity reduction or ITZ improvement.

**Figure 14:**
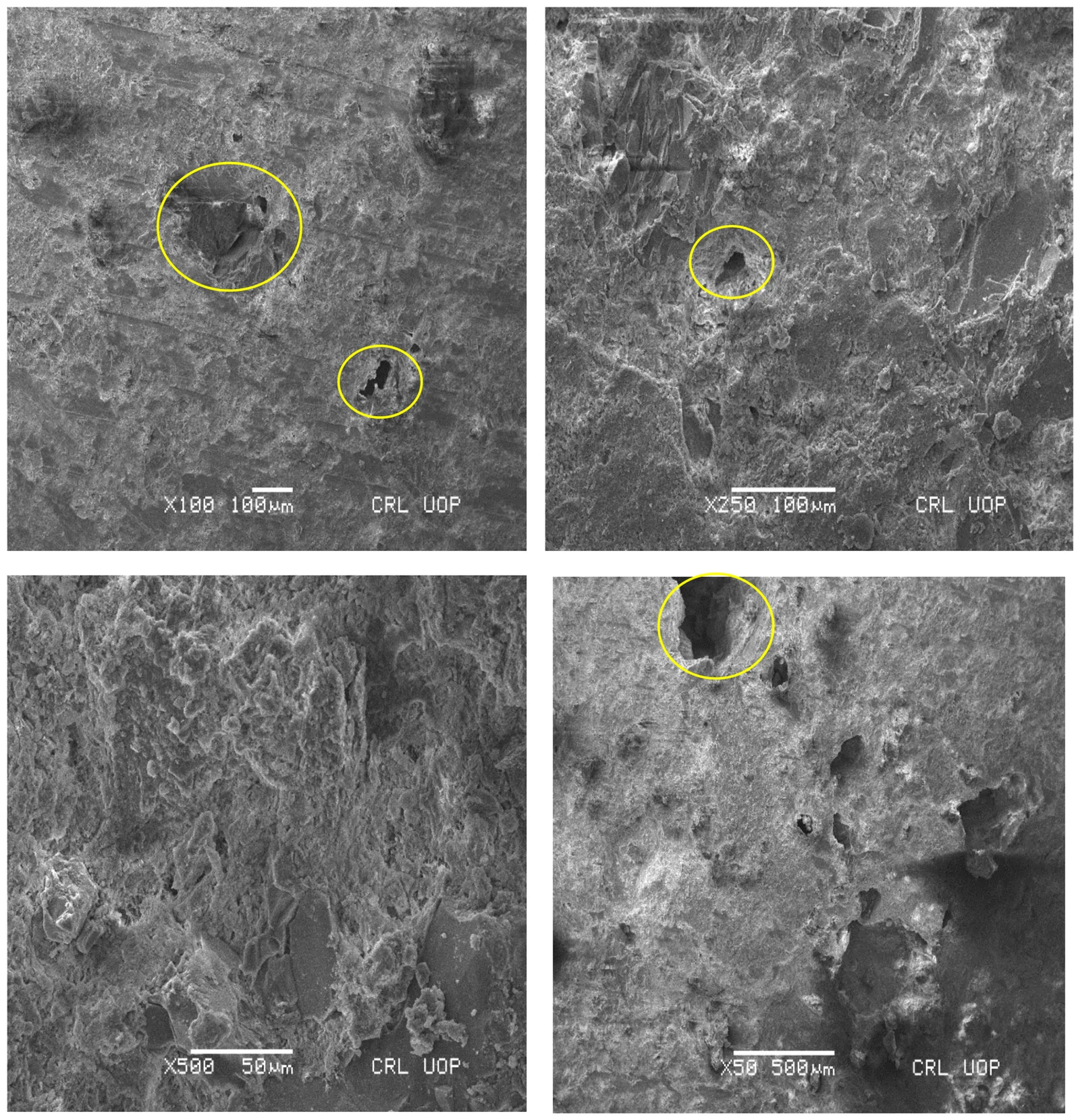
SEM of HCC-R30-B20 mix.

The SEM of HCC-R30-B20 exhibits a relatively dense and compact microstructure compared with the control mixture (Figure 14). Smaller pores are observed, which qualitatively suggests improved particle packing and reduced void connectivity. The reduction in large capillary voids observed in the selected SEM region suggests that the 30% RHA and 20% bentonite clay contribute to enhanced matrix densification through combined pozzolanic reaction and filling effects. Similarly, a previous study [25] noted that the small particle size of RHA contributes to improved packing density by filling micro-voids and enhancing the bonding within the cementitious mix. However, the SEM observations represent localized morphology and cannot be used to quantify the overall porosity.

Therefore, the microstructure appears more homogeneous, with a continuous gel matrix covering previously porous regions. The presence of bentonite clay, which possesses fine particle characteristics and high surface area, is evident. Therefore, it can provide nucleation sites for hydration products and promote refinement of pore structure. Consequently, discontinuities and microcracks are less pronounced compared with the control mix. Also, the ITZ appears denser and more integrated with the surrounding matrix. Therefore, this indicates improved adhesion between aggregate particles and the binder phase. The filler and nucleation effects of RHA and bentonite contribute to reduced porosity within the ITZ.

**Figure 15:**
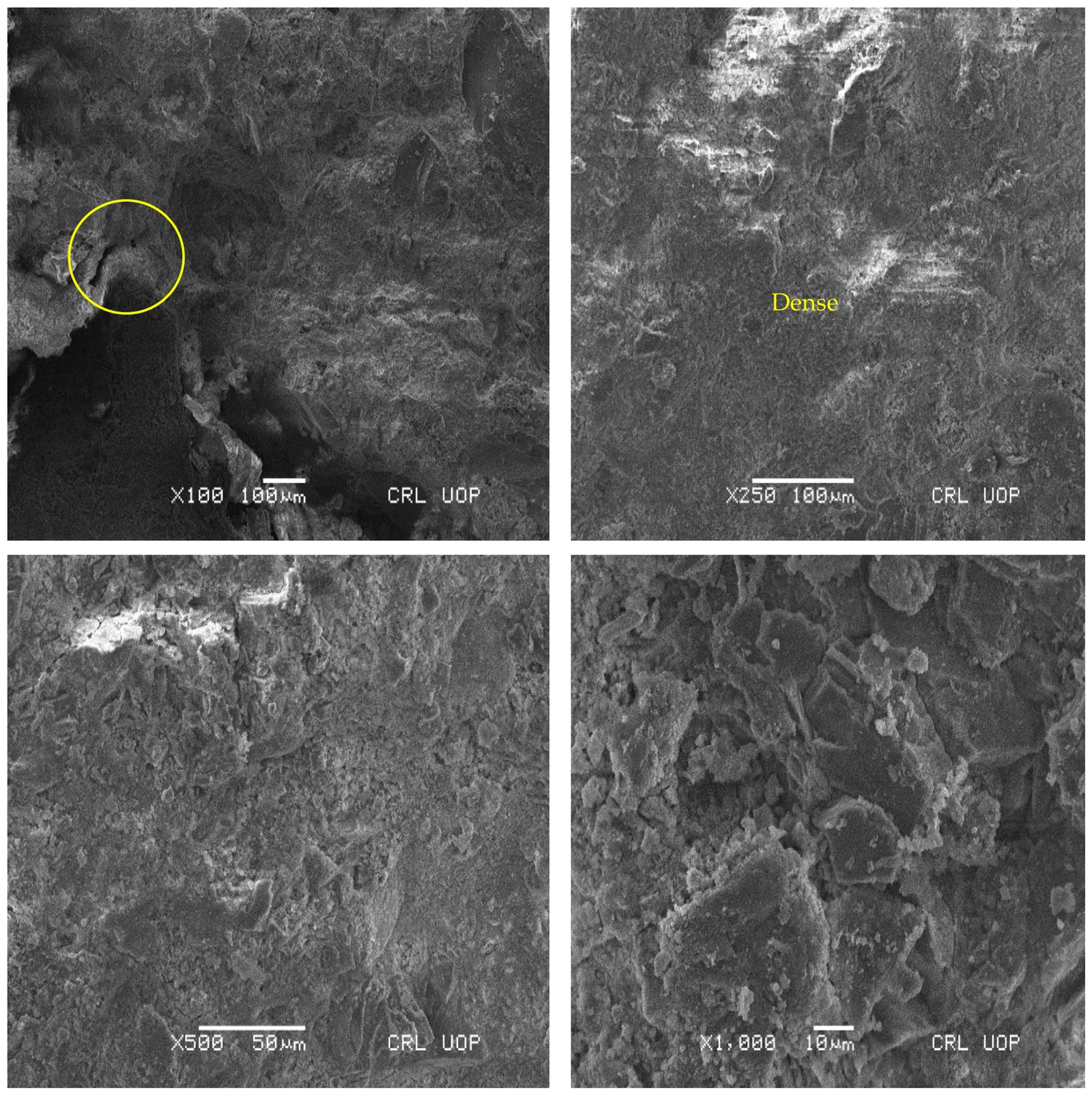
SEM of HCC-R30-Q20 mix.

The HCC-R30-Q20 SEM demonstrates a dense and refined microstructure as compared to the control mix (Figure 15). Therefore, it indicates the contribution of quartz powder (QP) as an inert micro-filler in improving particle packing density. The ITZ appears denser and less porous compared to the control mixture. A previous study also indicates that the enhancement in strength can be attributed to the pozzolanic nature of bentonite clay, which is rich in silica (SiO_2_). The fine particles of bentonite act as micro-fillers and occupy void spaces within the concrete mix [13]. Therefore, it demonstrates improved bonding between aggregate and paste matrix. The filler effect of quartz powder improves particle size distribution and promotes nucleation sites for hydration products, resulting in reduced microcracking and improved microstructural properties. However, more pores were observed in HCC-R30-Q20 as compared to HCC-R30-B20. Therefore, it is suggested that bentonite clay (BC) contributes more effectively to microstructural densification than quartz powder. The observations are consistent with the experimental mechanical results, which indicate that HCC-R30-B20 achieved higher compressive strength than HCC-R30-Q20. The comparatively denser microstructure and improved ITZ in HCC-R30-B20 enhance stress transfer efficiency and reduce crack propagation. However, the relationship between SEM morphology and mechanical performance should be interpreted cautiously because compressive strength is also affected by factors such as pore-size distribution, degree of reaction, binder chemistry, and bonding characteristics that may not be fully captured by localized SEM images. However, SEM images show only localized areas and compressive strength also depends on factors such as pore size, degree of reaction, and binder chemistry that cannot be fully observed in SEM analysis.

**Figure 16:**
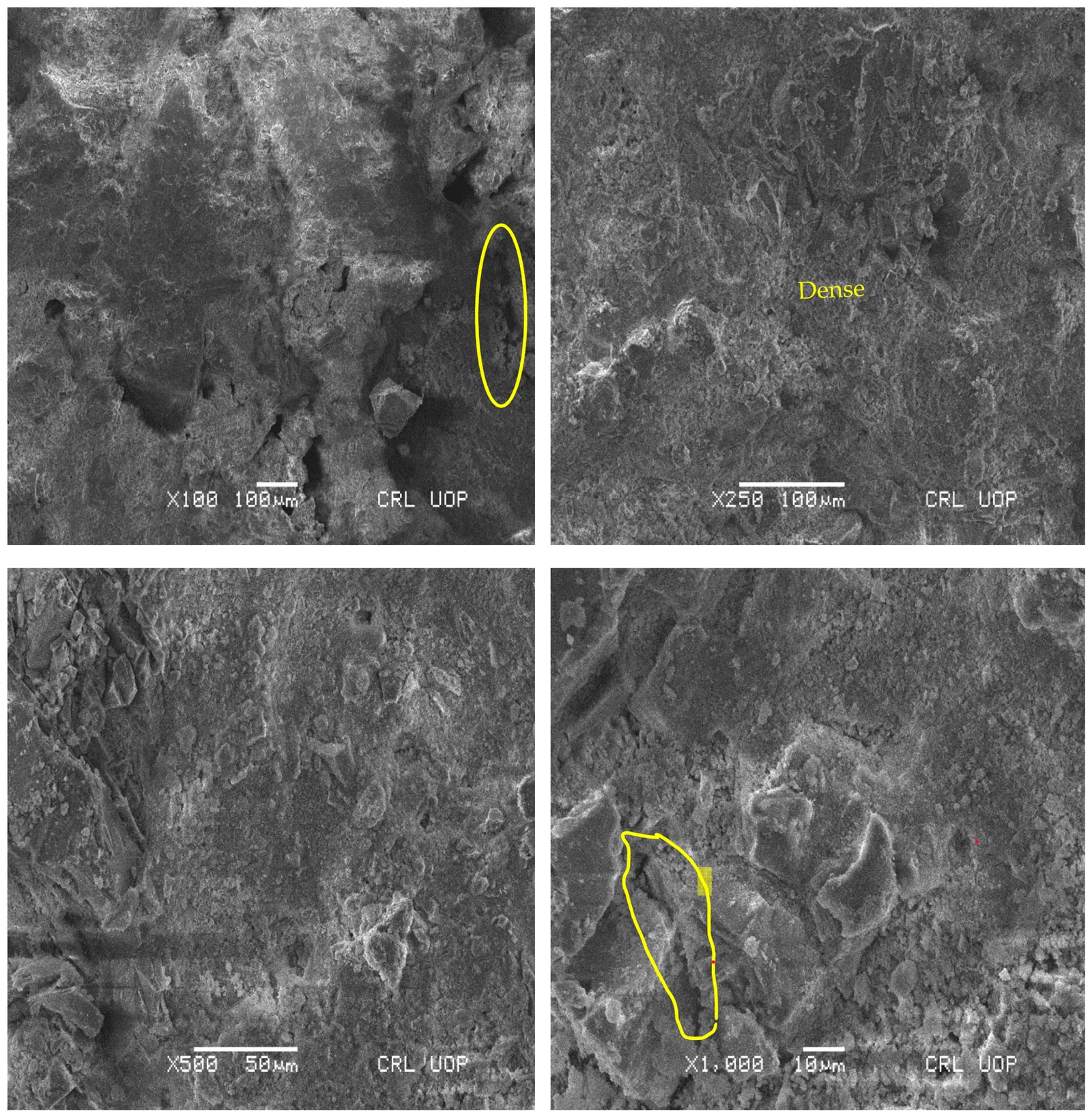
SEM of HCC-R20-Q20-B20 mix.

The SEM of HCC-R20-Q20-B20 shows a dense and refined microstructure (Figure 16), which is improved compared with the control mixture due to the synergistic interaction between RHA, QP, and BC. The combined presence of pozzolanic material (RHA), inert micro-filler (QP), and fine particle (BC) contributes to a more homogeneous structure. A dense gel matrix is clearly observed, demonstrating improved distribution of hydration products and better microstructural continuity. The BC enhances nanoscale filling ability, and QP improves particle size distribution. Therefore, together, they reduce capillary porosity and refine pore structure. Also, ITZ appears denser than the control mixture, suggesting improved bonding between aggregate particles and the binder mix. The ternary binder system promotes multiple nucleation sites, resulting in improved formation of CSH gel. However, the SEM analysis alone cannot confirm the formation of hydration products. In contrast, the SEM images of HCC-R20-Q20-B20 show a relatively dense matrix compared with the control mixture, but slightly less compact than HCC-R30-Q20 and HCC-R30-B20. Some localized pores and cracks are visible, which suggests that the ternary binder system does not produce the visually densest structure among the investigated mixes.

Therefore, the microstructural observations do not fully correlate with the mechanical performance results, where HCC-R20-Q20-B20 achieved the highest compressive strength. The difference can be attributed to the synergistic interaction between RHA, QP, and BC, which enhances the quality of hydration products even if it appears slightly less dense in SEM. The combined presence of pozzolanic reaction (RHA), micro-filler packing (QP), and fine particles (BC) can improve the chemical composition and stress transfer efficiency within the matrix. Furthermore, compressive strength is influenced not only by visible pores. It can also be influenced by pore size distribution, gel chemistry, degree of reaction, and ITZ bonding characteristics. Therefore, these aspects may not always be fully captured in localized SEM analysis. The findings highlight that microstructural features alone do not solely govern compressive strength. The SEM results are considered supportive qualitative evidence of microstructural differences rather than definitive quantitative evidence of porosity or ITZ improvement.

### 3.7. Energy Dispersive X-Ray (EDX) Analysis

**Figure 17:**
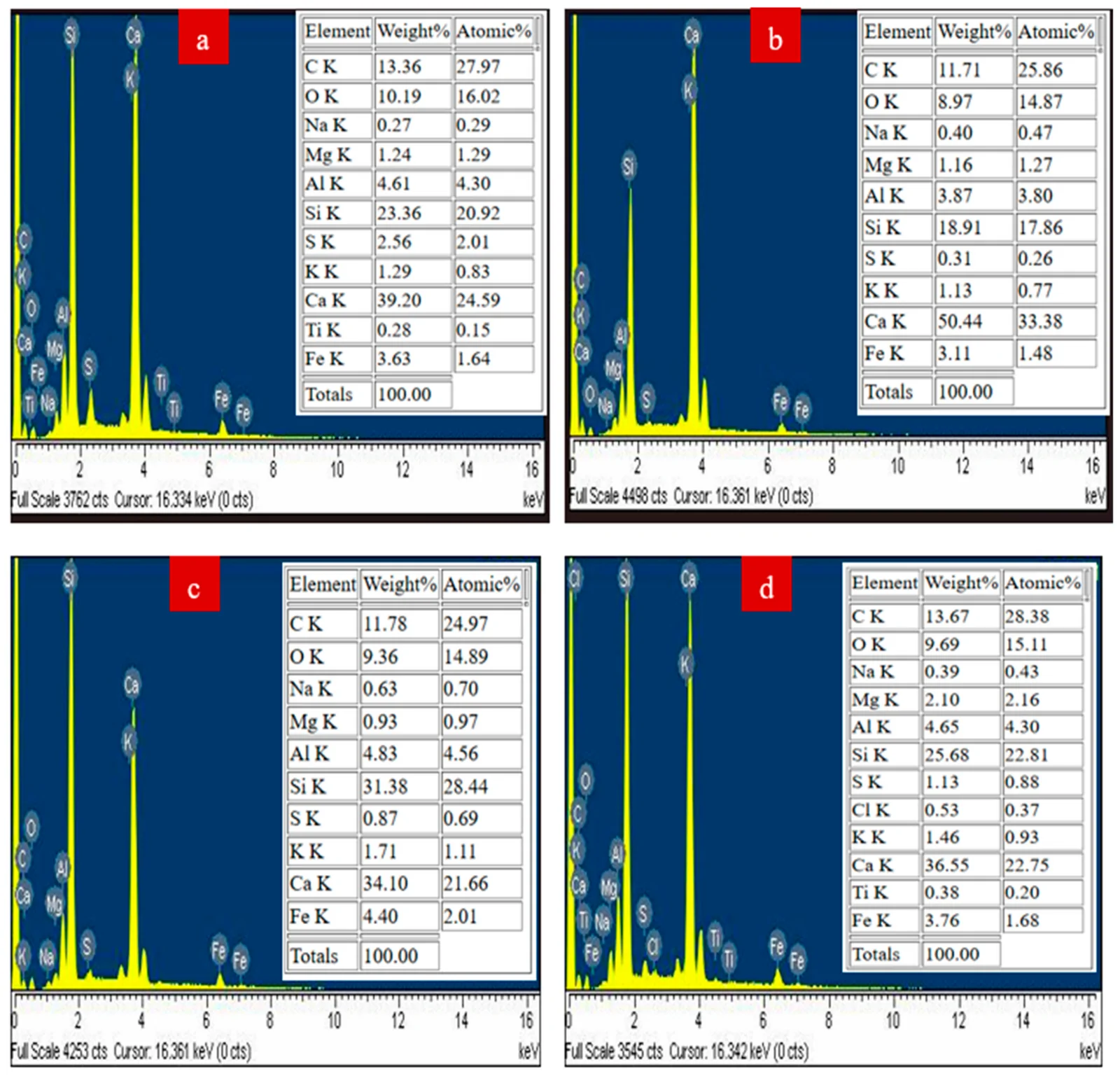
EDX analysis of the control and HCC mixes: (**a**) Control, (**b**) HCC-R30-B20, (**c**) HCC-R30-Q20 and (**d**) HCC-R20-Q20-B20.

The Energy Dispersive X-ray (EDX) analysis of the control mix (OPC) shows the presence of major elements typically associated with cement hydration products (Figure 17). The spectrum shows dominant peaks of calcium (Ca), silicon (Si), oxygen (O), aluminium (Al), and carbon (C). The high percentage of Ca (39.20%) suggests a Ca–Si-rich cementitious mix. The presence of Si (23.36%) and Al (4.61%) suggests the presence of silicon- and aluminium-bearing compounds. Oxygen (10.19%) is associated with oxide compounds forming the primary cement hydration products. Also, minor elements such as Mg, Fe, Na, K, S, and Ti are detected, which originate from cement clinker phases and supplementary mineral components. The presence of carbon (13.36%) may be attributed to carbonation effects. The EDX results suggest that the control mix is dominated by calcium-based hydration products.

The EDX spectrum of HCC-R30-B20 suggests the presence of major elements commonly associated with hydration and pozzolanic reaction products, including Ca, Si, Al, O, and C. The high Ca content (50.44%) and Si (18.91%) suggest the formation of calcium- silica hydration products such as CSH gel. The CSH gel plays a major role in strength development. The presence of Si (18.91%) and Al (3.87%) suggests the formation of calcium–aluminosilicate hydrate phases.

Compared with the control mix, the 30% RHA and 20% BC modify the chemical composition of the binder system. Bentonite clay, which contains reactive aluminosilicate minerals, can provide nucleation sites for hydration products and contributes to refinement of pore structure. The relatively high calcium content suggests that hydration reactions are still governed by calcium-based products, and the presence of silica from RHA contributes to secondary pozzolanic reaction, which can form additional CSH gel. Minor elements such as Mg, Fe, Na, K, and S originate from cement clinker phases and clay minerals. The reduced carbon content compared with the control mixture suggests a reduced carbonation effect. The elemental composition supports SEM observations showing a relatively dense microstructure and refined ITZ in HCC-R30-B20. The fine particle characteristics of bentonite can improve particle packing at the nano-scale level and enhance gel formation.

The EDX spectrum of HCC-R30-Q20 shows the presence of major elements associated with cement hydration and pozzolanic reaction products, primarily Si, Ca, Al, O, and C. A relatively high silica content (Si = 31.38%) is observed compared with the control and HCC-R30-B20 mixtures. The increased silica content can enhance the formation of additional CSH gel and contributes to improved mix densification. The calcium content (Ca = 34.10%) is lower than HCC-R30-B20 but sufficient to support hydration reactions and formation of calcium-based binding phases. The relatively higher Si/Ca ratio suggests a more silica-rich hydration environment, which is often associated with improved microstructural refinement and reduced pore connectivity. The presence of Al (4.83%) suggests aluminosilicate contribution from RHA and cement phases. Also, Quartz powder primarily acts as an inert micro-filler and fills micro-voids between cementitious particles. The filler effect enhances particle size distribution and provides additional nucleation sites for hydration products. Therefore, it leads to a refined pore structure and reduced microcracking. The improved distribution of hydration products contributes to the relatively dense microstructure observed in SEM analysis.

The EDX spectrum of HCC-R20-Q20-B20 can suggest the presence of major elements associated with hydration, including Ca, Si, Al, O, and C. The elemental composition shows a balanced proportion of calcium (Ca = 36.55%) and silicon (Si = 25.68%), suggesting the formation of CSH and calcium–aluminosilicate hydrate (CASH) gel phases. The presence of aluminium (Al = 4.65%) suggests the contribution of BC and RHA in forming additional aluminosilicate binding phases. The combined use of RHA, QP, and BC provides a multi-scale modification mechanism in which quartz powder improves particle packing density, bentonite clay enhances nano-scale filling ability, and RHA contributes reactive silica for secondary pozzolanic reaction. Minor elements such as Mg, Na, K, Fe, Ti, and Cl may originate from cement clinker phases and clay minerals, reflecting the complex mineralogical composition of the ternary binder system. SEM images indicate that HCC-R20-Q20-B20 may appear slightly less dense than HCC-R30-Q20. However, the EDX results suggest an optimized chemical composition that promotes stronger bonding phases. The presence of both silica-rich and alumina-rich components can enhanced the binding gel and improves microstructural properties. These findings support the mechanical performance results, where HCC-R20-Q20-B20 achieved the highest compressive strength among all mixes. The improved chemical composition can contribute to enhanced mix cohesion and improved ITZ bonding. Therefore, the compressive strength depends not only on visible pore structure but also on the quality and chemistry of hydration products.

### 3.8. Ca/Si Ratio

**Figure 18:**
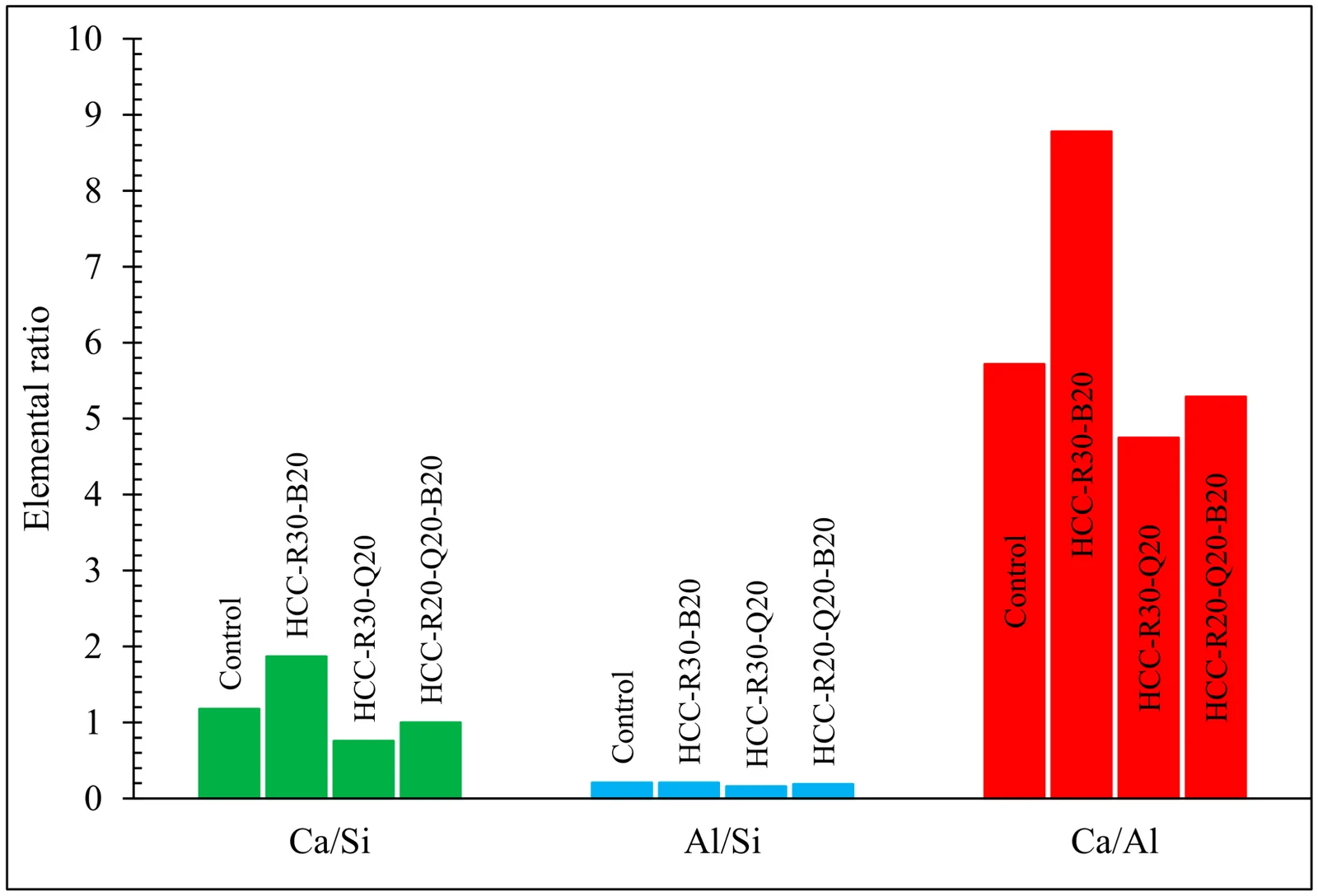
Elemental ratios of the different concrete mixes based on EDX analysis.

The Ca/Si ratio reflects the relative proportion of calcium to silica in concrete mix (Figure 18). The control shows the Ca/Si ratio associated with conventional OPC hydration products. Therefore, it is characterized by the coexistence of CSH gel and calcium hydroxide (CH). In contrast, modified HCC mixtures show variations in the Ca/Si ratio due to the silica-rich supplementary waste materials. HCC-R30-Q20 demonstrates a relatively low Ca/Si ratio (0.76) due to the silica contribution from quartz powder (QP) and rice husk ash (RHA). Therefore, it can promotes secondary pozzolanic reaction and enhances CSH formation. However, the HCC-R30-B20 mix shows a comparatively higher Ca/Si ratio. Therefore, it suggests increased calcium-rich phases associated with cement hydration. The ternary mixture HCC-R20-Q20-B20 shows a balanced Ca/Si ratio (1.0). Therefore, it suggests that the formation of C(A)SH gel improved structural strength and enhanced load transfer capability.

The control mixture exhibits an Al/Si ratio of 0.21, which is consistent with typical OPC hydration products where limited aluminium participation occurs mainly through partial substitution in CSH gel and formation of minor aluminate phases. The HCC-R30-B20 mixture also shows an Al/Si ratio of 0.21, indicating that the incorporation of B20 does not significantly alter the degree of aluminium. Therefore, the findings suggest that hydration products remain predominantly similar to conventional cementitious systems with comparable aluminosilicate participation. In contrast, HCC-R30-Q20 shows a lower Al/Si ratio of 0.16. This can be attributed to the silica contribution from QP and RHA. The increased availability of reactive silica can enhance the formation of silica-rich CSH gel with relatively lower aluminium. The shift suggest a more silicate-dominated gel structure. It is typically associated with increased chain length and improved gel densification. The ternary mixture HCC-R20-Q20-B20 shows Al/Si ratio of 0.19. The findings suggest a combined interaction between silica-rich and alumina-containing components, promoting the formation of C–(A)–S–H gel. SEM observations indicate that HCC-R30-Q20 exhibits the visually densest morphology. However, the elemental ratio analysis demonstrates that HCC-R20-Q20-B20 possesses a more optimized chemical composition, which is consistent with compressive strength. The synergistic interaction between the pozzolanic reaction of RHA, QP, and BC promotes the formation of efficient binding phases. Therefore, the SEM and EDX results are interpreted as complementary qualitative evidence rather than definitive proof. More detailed techniques such as XRD or FTIR would be needed for confirmation.

## 4. Conclusions

This study investigated the effects of RHA, QP, and BC on the fresh, mechanical, and water absorption characteristics of HCC. The following conclusions can be drawn:

- RHA, QP, and BC reduced the workability due to their high specific surface area, angular particle nature, and higher water absorption capacity. However, all HCC mixtures maintained acceptable slump values suitable for practical concrete applications.
- RHA, QP, and BC enhanced the compressive strength, with the ternary blend HCC-R20-Q20-B20 exhibiting the highest 28-day strength (22.1% increase compared with the control mix).
- RHA, QP, and BC affected the failure behavior and load-carrying capacity compared with the control mix.
- RHA, QP, and BC improved the tensile strength, with the highest value of 4.56 MPa achieved by HCC-R30-Q20 (18.4% increase compared with the control mix.
- RHA, QP, and BC reduced water absorption, with the lowest water absorption of 4.0% achieved by HCC-R30-Q20 (23.1% reduction compared with the control mix).
- RHA, QP, and BC caused only a slight reduction in density, with values ranging from 2380 to 2408 kg/m^3^ compared with 2415 kg/m^3^ for the control mix (all mixtures remained within the normal-weight concrete range).
- The SEM analysis suggests that RHA, QP, and BC refined the microstructure, reduced porosity, improved ITZ quality, and enhanced matrix densification.
- The EDX and elemental ratio analyses suggest that RHA, QP, and BC influenced the chemical composition as reflected by variations in the Ca/Si and Al/Si ratios.

The findings demonstrate that the combined RHA, QP, and BC shows potential for reducing cement consumption with enhanced mechanical performance through enhanced particle packing, pozzolanic activity, and microstructural refinement. However, the limited number of specimens restricts the statistical reliability and generalizability of the findings. Further studies with larger datasets and statistical analysis are recommended for validation.

## Figures and Tables

**Table 1:** Mix proportions of developed HCC.

Material	Control	HCC-R30-B20	HCC-R30-Q20	HCC-R20-Q20-B20
Cement (kg/m^3^)	420	210	210	168
Rice Husk Ash (kg/m^3^)	0	126	126	84
Quartz Powder (kg/m^3^)	0	0	84	84
Bentonite (kg/m^3^)	0	84	0	84
Fine Aggregate (kg/m^3^)	550	550	550	550
Coarse Aggregate (kg/m^3^)	1020	1020	1020	1020
Water (kg/m^3^)	170	170	170	170
Superplasticizer (kg/m^3^)	2.1	2.1	2.1	2.1

Nomenclature: R, Q, and B represent RHA, QP, and BC, respectively. The numbers indicate their percentage replacement of the total binder content (420 kg/m^3^). Accordingly, 30% and 20% replacement correspond to 126 kg/m^3^ and 84 kg/m^3^, respectively.

**Table 2:** Testing standards details.

Property	ASTM Standard	Reference
Slump	ASTM C143/C143M	[16]
Density	ASTM C642	[17]
Compressive strength	ASTM C39/C39M	[18]
Split tensile strength	ASTM C496/C496M	[19]
Water absorption	ASTM C642	[17]

## Data Availability

Data are contained within the article. The original contributions presented in this study are included in the article. Further inquiries can be directed to the corresponding author. The authors used Grammarly software to improve the grammar and language of the manuscript.
